# Using digital tools in the recruitment and retention in randomised controlled trials: survey of UK Clinical Trial Units and a qualitative study

**DOI:** 10.1186/s13063-020-04234-0

**Published:** 2020-04-03

**Authors:** Amanda Blatch-Jones, Jacqueline Nuttall, Abby Bull, Louise Worswick, Mark Mullee, Robert Peveler, Stephen Falk, Neil Tape, Jeremy Hinks, Athene J. Lane, Jeremy C. Wyatt, Gareth Griffiths

**Affiliations:** 1 National Institute for Health Research, Evaluation, Trials and Studies Coordinating Centre (NETSCC), University of Southampton, Southampton, SO16 7NS UK; 2 Southampton Clinical Trials Unit, University of Southampton and University Hospital Southampton NHS Foundation Trust, Southampton General Hospital, Southampton, SO16 6YD UK; 3 NIHR RDS (Research Design Service) South Central Level C (805), South Academic Block, Southampton General Hospital, Tremona Road, Southampton, SO16 6YD UK; 4 NIHR Clinical Research Network Wessex, 7, Berrywood Business Village, Tollbar Way, Hedge End, Southampton, SO30 2UN UK; 5 Bristol Cancer Institute, Horfield Road, Bristol, BS2 8ED UK; 6 Southampton General Hospital, University Hospital Southampton NHS Foundation Trust, Tremona Road, Southampton, SO16 6YD UK; 7 University of Southampton, University Road, Highfield Campus, Southampton, SO17 1BJ UK; 8 Bristol Randomised Trials Collaboration, Bristol Medical School, University of Bristol, Bristol, BS8 2PS UK; 9 Wessex Institute, University of Southampton, Southampton, SO16 7NS UK

**Keywords:** Digital tool, Participant recruitment, Participant retention, Qualitative, Survey, Clinical trials unit

## Abstract

**Background:**

Recruitment and retention of participants in randomised controlled trials (RCTs) is a key determinant of success but is challenging. Trialists and UK Clinical Research Collaboration (UKCRC) Clinical Trials Units (CTUs) are increasingly exploring the use of digital tools to identify, recruit and retain participants. The aim of this UK National Institute for Health Research (NIHR) study was to identify what digital tools are currently used by CTUs and understand the performance characteristics required to be judged useful.

**Methods:**

A scoping of searches (and a survey with NIHR funding staff), a survey with all 52 UKCRC CTUs and 16 qualitative interviews were conducted with five stakeholder groups including trialists within CTUs, funders and research participants. A purposive sampling approach was used to conduct the qualitative interviews during March–June 2018. Qualitative data were analysed using a content analysis and inductive approach.

**Results:**

Responses from 24 (46%) CTUs identified that database-screening tools were the most widely used digital tool for recruitment, with the majority being considered effective. The reason (and to whom) these tools were considered effective was in identifying potential participants (for both Site staff and CTU staff) and reaching recruitment target (for CTU staff/CI). Fewer retention tools were used, with short message service (SMS) or email reminders to participants being the most reported. The qualitative interviews revealed five themes across all groups: ‘*security and transparency*’; ‘*inclusivity and engagement*’; ‘*human interaction*’; ‘*obstacles and risks*’; and ‘*potential benefits*’. There was a high level of stakeholder acceptance of the use of digital tools to support trials, despite the lack of evidence to support them over more traditional techniques. Certain differences and similarities between stakeholder groups demonstrated the complexity and challenges of using digital tools for recruiting and retaining research participants.

**Conclusions:**

Our studies identified a range of digital tools in use in recruitment and retention of RCTs, despite the lack of high-quality evidence to support their use. Understanding the type of digital tools in use to support recruitment and retention will help to inform funders and the wider research community about their value and relevance for future RCTs. Consideration of further focused digital tool reviews and primary research will help to reduce gaps in the evidence base.

## Background

Recruitment of participants to, and their retention in, randomised controlled trials (RCTs) is a key determinant of research efficiency, but it can be challenging [1]. Reviews of clinical trials funded by the UK Medical Research Council (MRC) and the National Institute for Health Research (NIHR) Health Technology Assessment (HTA) programme [2] have shown that the proportion of trials achieving their original recruitment target was in the range of 31%–56%, and 79% of trials achieving at least 80% of the final target sample size (119/151, trials reporting from 2004 to April 2016) [3, 4]. Although the literature seems to suggest that there have been improvements over time, a recent study found that 44% of selected trials failed to reach their target [3, 5, 6]. Despite the vast amount of literature on strategies to improve recruitment and retention in clinical trials, the quality of the evidence is lacking [5–10]. The recently updated Cochrane Review on strategies to improve recruitment to RCTs found only three studies with a high Grading of Recommendations Assessment, Development and Evaluation (GRADE) rated evidence [6]. Given the lack of high-quality evidence and certainty around resource-intensive techniques to improve recruitment and retention, trialists and Clinical Trials Units (CTUs) are increasingly exploring the value of digital tools as a more viable option to identify, recruit and retain participants. Existing literature investigating the use of digital technology/tools for recruitment to and retention in clinical trials has mainly focused on:

- Eligibility: searches and interactive medical record tools to support clinicians screening participants [11];
- Recruitment: trial websites, social media and email campaigns to engage with the broader public [12–15];
- Retention: emails, interactive websites, text messages or apps to retain participants enrolled in trials and help them adhere to the trial intervention [9, 10, 16–18].

In theory, the use of digital tools should reduce research costs and speed up the delivery of results, improve recruitment rates and reduce the recruitment of ineligible participants (around 6% in one study [11]). However, selecting an appropriate digital tool for a trial is challenging because few have been evaluated rigorously. More challenging perhaps is the use of different success metrics to understand how and where digital tools add value to the recruitment and retention of participants in trials, e.g. reduced screening time, improved coverage of recruitment or percentage of participants recruited. Given the lack of evidence, there is a need to explore which success metrics are useful to determine which digital tools (and what features of the tools) are most relevant to stakeholders and encourage a wider uptake of the use of effective digital tools.

One systematic review, on databases to improve trial recruitment, located only nine studies using reasonably robust methods out of the 101 eligible studies [19]. It concluded that databases could reduce the time taken to screen participants, improve participant coverage and actual recruitment rates by between 14%, though four of the five studies making these measurements used an uncontrolled before–after design and the fifth was confounded. This suggests a need to assemble, map and critically appraise the evidence base for these digital tools to determine their value and benefit for improving recruitment and retention rates. Only then can we confidently advise on the wider use of such digital tools by trialists or on the need for further primary research.

The aim of this study was to answer the following two questions: (1) What are the digital tools that could help identify, recruit or retain participants in trials are used in UK CTUs? and (2) What performance characteristics do trialists and CTUs require of digital tools for them to be judged useful?

## Methods

The research reported here was part of a broader NIHR-funded project ‘User-focused research to identify the benefits of innovative digital recruitment and retention’. There were three phases in the main study:

- Phase 1: Scoping of searches and a survey of NIHR funder staff to determine what digital tools are currently being used by funded clinical trials.
- Phase 2: (1) A survey of CTUs on their experiences of digital tools; (2) the development of a logic model to help classify the digital tools into generic categories and identify potential outcome measures for Phase 3 and future primary studies; (3) qualitative interviews with key stakeholders to identify the characteristics of digital tools that they would judge useful and potential disadvantages of these tools.
- Phase 3: A systematic mapping exercise to identify and describe studies of the effectiveness and accuracy of digital tools for recruitment and/or retention in RCTs [20].

This paper reports on the Phase 1 and Phase 2 survey of CTUs and qualitative interviews. The other aspects of this project have been submitted elsewhere [20].

To ensure quality assurance, a Project Management Team and Project Board were created. The role of the Project Management Team included the day-to-day running of the project and the review and sign-off of all study documentation and analysis. The role of the Project Board was to oversee the work of the Project Management Team to ensure delivery of the project and adherence to the ethically approved research methods.

### Phase 1: Scoping of searches and a survey of funder staff

A scoping exercise was conducted to explore the range of digital tools used within trials to increase the efficiency of recruitment and retention in trials. This consisted of a review of published and grey literature (PubMed and Google Scholar) using a variation of key words and phrases:

- Digital / tools to improve study recruitment / retention in trials / clinical trials
- Digital platforms for trial recruitment
- Retention in research studies / digital platforms in use

To avoid duplication with the systematic mapping exercise conducted in Phase 3, this was limited to: Recruitment; Retention; Clinical trials; and Study design. A snowball approach was used to retrieve relevant literature. The online search was conducted in December 2017 and the results were shared with the project team to inform the content of the CTU survey questions and qualitative interview frameworks. The scoping exercise was not systematic (systematically applied a search criteria or systematic methods for replication). The purpose of the literature review was to inform the CTU survey and the qualitative interviews.

An online survey (using Google forms) was sent to NIHR *Evaluation, Trials and Studies Coordinating Centre* (NETSCC) research management staff in February 2018 to understand how many funded trials have used or are using digital tools across four of the NIHR research programmes (Efficacy and Mechanism Evaluation [EME], Health Services and Delivery Research [HSDR], Health Technology Assessment [HTA] and Public Health Research [PHR]). Examples of relevant digital tools were provided to help inform the research manager’s review of their portfolio of funded research projects (Additional file 1). A reminder was sent to NETSCC staff and the deadline was extended by 1 week with the option of discussing the survey with a member of the research team.

### Phase 2: CTU survey

Preliminary findings from Phase 1 were used to inform and develop the CTU survey. This survey (SurveyMonkey) was sent to the Directors of all UK Clinical Research Collaboration (UKCRC)-registered CTUs, via email circulation from the UKCRC administrator, during March–May 2018. The Directors were asked to disseminate this to the most relevant CTU staff member (e.g. Head of Trial Management / Operations). A webinar was conducted during March 2018 to help explain the nature of these digital tools and increase response and completion rate and a follow-up reminder email was sent to all participating CTUs via the same mechanism as the initial invitation. A definition of a digital tool and examples of digital tools were provided and CTUs were asked to list up to five recruitment tools and five retention tools they have experience of, and then to expand on up to two tools that had impressed them within each category and about one of either kind that they have experienced problems with. The questions focused on the performance characteristics related to the digital tools that are currently being used to identify, recruit and retain participant within CTU trials. The salient performance characteristics were: recruitment barrier for which the tool is a solution; its source (commercial, in-house, academic); the study context (was there a specific disease area, type of study and population). Costs were specifically not asked for due to the perceived difficulty in answering this questions; however, ease of configuration was asked (i.e. did an expert need to build it or could the CTU do it themselves). The last questions focused on estimated effectiveness and reason for and to whom (Additional file 2). The survey results were categorised, discussed and verified with the project team.

### Phase 2: Qualitative interviews

The sampling of participants was purposive due to the availability of resources, participant availability and the number of participants required to reach data saturation (the project team agreed on three participants per stakeholder group and if additional participants were required they would be approached and invited on a case-by-case basis). The project team were responsible for the eligibility criteria who work in clinical trials settings, ethics committee representatives and researchers from the NIHR. The potential sample of participants were chosen on a case-by-case basis (in agreement and consensus with project team members) from a broad range of professional backgrounds with diverse experiences of recruitment and/or retention in clinical trials. This sampling approach identified research professionals with appropriate trial recruitment experience based on those known to the project team members and recommendations from the Principal Investigator. A list of contact details for each stakeholder group for eligible participants was developed which included staff in medical research funding organisations and charities, individuals working on trials in primary and secondary care, and research participant representatives (Table 1). For the trialists group, those who participated in the CTU survey were asked if they would be willing to participate in an interview.

**Table 1:** Stakeholders for qualitative interviews

Stakeholder groups (number of participants)	Example of the type of roles held	Eligibility criteria (involvement in one or more of)
Trialists in CTUs (*n* = 3) in primary/secondary care	Trialists and recruiters	Trial recruitment
		Management of trials (CTU staff)
Research practitioners in primary care (*n* = 3)	Principal Investigators	Leading funding applications
Research funding bodies (*n* = 3)	Funding committee members / Charities	Contribute to decision-making of funding allocation from charities or NIHR
Ethics committees and Health Research Authority (*n* = 3)	Ethics committee members	Contribute to decision-making on research ethics
Research participant/patient representatives (*n* = 4)	Research participant representatives	Experience of participating in trials

*CTU* Clinical Trials Unit, *NIHR* National Institute for Health Research

A potential sample of participants were selected before sending out an invitation by email to participate. If any potential participants declined the invitation, further participants were chosen from the groups that were identified at the start of the sampling process.

The participant representatives were chosen through existing local patient groups associated with the local CTU (Southampton). The initial plan was to engage with participant representatives through focus groups but we were unable to recruit the appropriate number of participants to take part in the time period allowed to conduct these (none of the participants agreed to participate in the focus group). As a result, we offered telephone interviews as an alternative and we were able to recruit the proposed number of participant representatives (*n* = 4) (as outlined in the ethics application if focus groups were not feasible, interviews will be offered to the participant representatives). The sample method applied to the stakeholder groups were therefore used for the participant representative group.

The decision to approach the agreed sampling groups was based upon consensus from discussions with the project team to provide sufficient data to answer the research question and provide wide coverage of the use of digital tools. All potential participant groups, including the research participant representatives responded to an ‘invitation to volunteer’ from the study researcher through the CTU (email from the current project Principal Investigator). A participant information sheet was sent with the invitation, asking the recipients of these invitations to approach the researcher directly via email or phone to indicate their interest in participating in an interview. They were invited via email to participate in an interview at a mutually convenient time and date. Email reminders were sent after initial contact of the original invitation.

Participants engaged in a 45-min, semi-structured telephone interview (Additional file 3). The professional group were not specifically asked about the acceptance of digital tools and the participant representative stakeholder group were not specifically asked about the intended outcomes, participant perspective or their awareness of the evidence. The interview framework was developed from the CTU survey findings, and interviews were undertaken during May–June 2018. Each participant’s identity remained anonymous in all reports and identifying data were password-protected and only accessible by the research team (outlined in the General Data Protection Regulation [GDPR] May 2018).

An inductive approach was used to establish clear links between the interview framework for data collection and the summary of findings from the raw data (i.e. the interviews). Two researchers conducted dual coding for quality assurance measures. Data saturation was reached when no new constructs, categories or themes emerged from the interview data. A content analysis was conducted to provide a summary of the interview data. Both processes were conducted using Microsoft Excel 2016 (Microsoft Corporation). The theoretical framework for the interviews and schedules are available (Additional files 3 and 4, respectively). Ethics approval was granted by the Faculty of Medicine Ethics Committee, University of Southampton (Submission Number 32140) and the qualitative study was conducted to provide the ‘Outcomes’ and ‘Methodology’ of the PICO-M (Population, Intervention, Comparator and Outcomes Methodology) that shaped the systematic mapping exercise and the study eligibility criteria.

## Results

### Phase 1: Scoping of searches and a survey of funder staff

The scoping exercise revealed 46 examples of digital tools, approaches or services: 23 online tools and applications; 16 clinical trial companies offering recruitment and retention services; and seven online forums or companies facilitating patient involvement. The preliminary survey was sent to all 28 NETSCC Research Managers in February 2018. Thirteen (46%) staff completed the survey, yielding 26 examples of NIHR-funded trials using digital tools (either for recruitment or retention; Table 2) across four NIHR funding programmes (Table 3). Only a limited number of trials provided a detailed account of the digital tools used to help enhance the recruitment and/or retention of participants.

**Table 2:** Breakdown of the type of NIHR funding schemes where a digital tool was identified in survey of NETSCC monitoring staff

	n (%)
*NETSCC programme*	
EME	4 (15.4)
HTA	16 (61.5)
HSDR	5 (19.2)
PHR	1 (3.9)
*Work stream*	
Commissioned	14 (53.8)
Researcher-led	11 (42.3)
Themed call	1 (3.9)

*EME* Efficacy and Mechanisms Evaluation, *HSDR* Health Service and Delivery Research, *HTA* Health Technology Assessment, *NIHR* National Institute for Health Research, *PHR* Public Health Research

**Table 3:** Types of digital approaches used in NIHR studies from funding staff (multi-responses)

Example of digital tools used	n (%)
Social media (e.g. Facebook, Twitter)	6 (23.1)
Text messaging	6 (23.1)
Online project websites	6 (23.1)
Named online services (e.g. Quintet)	3 (11.5)
Software / Apps (e.g. bespoke for intervention such as StopApp digital intervention or unnamed digital software platform reported)	4 (15.4)
Limited information reported	5 (19.2)

*NIHR* National Institute for Health Research

### Phase 2: CTU survey

Twenty-four (46%) of 52 UKCRC CTUs responded to the survey; 6 (25%) stated no prior tool use and 18 (75%) reported five main areas of digital tool experience in recruitment (Table 4) and retention **(**Table 5). In the 24 responses, 41 recruitment and 29 retention tools were mentioned, and CTUs provided detailed answers for 22 recruitment tools (Table 6) and 15 retention tools (Table 7). The most frequently mentioned tools were related to database screening tools (e.g. Clinical Practice Research Datalink [CPRD] and in-house built screening tools (e.g. Medical Information Systems [EMIS]) (19/41, 46%). Of these 19 database screening tools, 10 (45%) responders provided additional information, of which 7 (70%) respondents felt these database screening tools were the most effective. The reason (and to whom) these tools were considered effective was in identifying potential participants (for both Site staff and CTU staff) and reaching recruitment target (for CTU staff/CI). The reasons for using these tools and most frequently mentioned barriers were to identify patients quicker and thus save time, poor record keeping, reminders to recruit and so on (6/10, 60%). Other performance characteristics of these tools were: they were either commercial (4/10, 40%) or built in-house (5/10, 50%) and were mainly used across all disease areas (6/10, 60%); they were not specific to the needs of any care group (e.g. children, frail, adults with learning disabilities, etc.) (9/10, 90%); and for all study types (7/10, 70%). To configure these tools, an expert was required in most cases (7/10, 70%). Although social media was only mentioned by 6/22 (27%) responders, and was the most frequently mentioned tool for ‘increasing trial reach to participants’ (5/6, 83%), the estimated effectiveness varied considerably, with only 17% stating ‘very effective’ (1/6).

**Table 4:** Number of digital tools, by category that CTUs mentioned they have experience of in relation to recruitment (including identification)

Recruitment tools	n (%)
Database tool (CPRD - Clinical Practice Research Datalink, in-house built tool, disease registry)	19 (46)
Social media (e.g. Facebook, Twitter, YouTube)	11(27)
Trial websites	7 (17)
ISRCTN, clinicaltrials.gov	3 (7)
Other – in-house	1 (2)

*CTU* Clinical Trials Unit, *CPRD* Clinical Practice Research Datalink

**Table 5:** Number of digital retention tools that CTUs mentioned they have experience of in relation to retention, broken down by category

Retention tools	n (%)
SMS / email reminders	17 (59)
Study websites	3 (10)
Apps and web-based data collection	3 (10)
Social media	2 (7)
Other – in-house	4 (14)

*CTU* Clinical Trials Unit

**Table 6:** Number of digital tools in relation to recruitment that CTUs mentioned they have experience of, broken down by category

Recruitment tools	n (%)
Database tool (Clinical Practice Research Datalink [CPRD], in-house built tool, disease registry)	10 (45)
Social media (e.g. Facebook, Twitter, YouTube)	6 (27)
Trial websites	4 (18)
ISRCTN, clinicaltrials.gov	1 (5)
Other – in-house	1 (5)

*CTU* Clinical Trials Unit, *CPRD* Clinical Practice Research Datalink

**Table 7:** Number of digital retention tools CTUs mentioned that had impressed them, broken down by category

Retention tools	n (%)
SMS / email reminders	10 (67)
Study websites	0 (0)
Apps and web-based data collection	0 (0)
Social media	1 (7)
Other – in-house	4 (18)

*CTU* Clinical Trials Unit

Fewer retention tools were mentioned by the responders, with almost half reporting the use of SMS/email reminders (17/29, 59%) and most CTUs choosing to expand on the use of SMS/email reminders than any other tool (10/15, 67%). The majority of the SMS/email reminder programmes were developed in-house and bespoke (7/10, 70%), used for more than one study (9/10, 90%) and required an expert to develop the tool (7/10, 70%). However, the certainty surrounding effectiveness varied and only one CTU stated their tool to be very effective (10%).

No CTU mentioned a tool that had caused problems for recruitment or retention.

Table 8 provides a list of potential digital tools to support recruitment and retention tasks that was developed from the survey results.

**Table 8:** List of potential digital tools to support recruitment and retention tasks

Task	Target	Possible tools
Publicise a trial	Recruiters (site and CTU trial staff)	Social media, email campaign
Identify possible patients for a trial offline	Recruiters (site staff only)	Database screening (e.g. CPRD) Trial eligibility checklist on trial website
Identify patients for trial during consultation	Recruiters (site staff only)	Automated flag based on EPR
Ensure patient really was eligible for trial when recruited	Recruiter (site staff only)	EPR database check on entry
Incentivise recruiters	Recruiters (site and CTU trial staff)	Automated league table, lottery for recruiters Simplified trial recruitment workflow Online patient information/video, etc.
Raise public awareness about trials in general	Public / patients	Social media, email campaigns
Help patients find a specific trial	Public / patients	clinicaltrials.gov, trial website; Google ads or pop up on disease website
Improve public understanding of a specific trial	Patients	Trial website eConsent video, animated patient information leaflet Web chat with trial nurse App (software) providing tailored information for patient

*CPRD* Clinical Practice Research Datalink, *CTU* Clinical Trials Unit, *EPR* electronic patient records

### Phase 2: Qualitative interviews

Sixteen interviews were conducted across five stakeholder groups during a 2-month period (May–June 2018). The content analysis based on the interview framework topics (Table 9) revealed that the two most commonly discussed areas across all stakeholders were the barriers, challenges and benefits of using digital tools. There were some necessary differences between the interview frameworks for the professional and participant representative stakeholders; these are identified in Table 9 under Not Applicable.

**Table 9:** Content analysis of responses from the five stakeholder groups

Stakeholder group →	Trialist secondary care	Primary care	Ethics committee	Funding committee	Participant representation	Totals
**Interview framework topics ↓**						
Generic statements	3	3	3	3	NA	12
Benefit	2	2	0	3	4	11
Intended outcomes of digital tools	1	0	0	1	NA	2
Acceptance	NA	NA	NA	NA	4	4
Challenges/barriers	2	3	3	3	4	15
Participant perspective	2	2	2	1	NA	9
Awareness of evidence	2	1	3	2	NA	8
Funding issues	2	1	0	2	1	6
Ethics	2	2	3	0	2	9
GDPR / Security	2	1	1	1	4	9
PPI	3	1	2	1	NA	7
Evidence	2	2	0	3	NA	7
Tools used	3	3	0	1	NA	7
Knowledge of digital tools	NA	NA	NA	NA	4	4

*GDPR*, *NA* not applicable, *GDPR* - General Data Protection Regulation, *PPI* - Patient and Public Involvement

In order to help shape the analysis and results, we captured key headlines for each stakeholder group by the topic areas covered in the interviews to demonstrate the variability and/or similarities between stakeholders. The initial analysis revealed nine themes but these were then merged to five themes: security and transparency; inclusivity and engagement; human interaction; obstacles and risks; and potential benefits of digital tools (shown in Table 10).

**Table 10:** Summary of themes identified

**Theme 1: Security and transparency** - Security and legitimacy of information and data [sharing] - Efficiency and transparency of information and data **Theme 2: Inclusivity and engagement** - Equity and inclusion of populations - Recognition of the ability / inability to use digital tools **Theme 3: Human interaction** - Trade-off between human face to face and digital tools - Lose sight of human interface and the importance of face-to-face connection **Theme 4: Obstacles and risks** - Obstacles preventing the use of digital tools (e.g. evidence, barriers, solutions) - Risk of technology overload **Theme 5: Potential benefits** - Unknown potential for the use of digital tools (e.g. evidence) - Reducing the burden on participants (e.g. convenience, time)	

### Theme 1: Security and transparency

Although there was unified acceptance of the use of digital tools, all participants felt that they should not lose sight of the issues and barriers around security, legitimacy and transparency of data. Concerns around the risk of data breach, suitable digital platforms and anonymization of data were key considerations and concerns to all participants.

‘*There was a fear … there still is a fear … where is it going to, who is holding this information? … If you’re throwing out an invitation you have to know where you are throwing it.*’ (ID5 – Ethics Committee member)

For participant representatives, there was greater concern about how and where data are stored and how to legitimise the point of initial contact.

‘*… I would Google the email address, which I do quite frequently now, with all the GDPR stuff … I would look to see if it was a bona fide email, and go in via the Internet rather than something that has perhaps been linked essentially … I think as long as you know that it is a … it has got governance, I suppose when you are looking at**clinicaltrials.gov**, you know it is part of the government framework.*’ (ID4 – Participant representative)

### Theme 2: Inclusivity and engagement

Adopting the use of digital tools across different types of populations was considered essential for all participant groups. This not only focused on the perspective of the individual but also the availability and acceptance of using a digital device, including using a digital interface (e.g. downloading an application to a mobile device). The key findings from stakeholder professionals mainly focused on people’s ability to use digital tools and the appropriateness for some participant groups.

‘*Some patients, they’re quite happy having everything emailed, others want telephone calls … its different for everyone so I think you just have to be flexible.*’ (ID10 – Practitioner in Primary Care)

‘*Potential to exclude people based on their ability to use these tools … you might exclude people who don’t have a smart phone …*’ (ID3-Practitioners in Primary Care)

‘*Approaching people by a different route is potentially a way of providing information to people that they wouldn’t otherwise be given through the typical kind of health professionals’ route. It opens up an avenue for actually getting greater dissemination about research opportunities.*’ (ID1 – Trialist)

The participant representatives were generally accepting of digital tools, although they felt that careful consideration is needed when applying these tools across different participant groups. The consensus was more focused on use of data, and accessibility to participate in clinical trials.

‘*I just think if it’s going to help someone else, then they’re welcome to it … No, that doesn’t bother me … I would be more worried about things like my bank account than, you know, someone might know what things that I’ve had or what I’m doing.*’ (ID2 – Participant representative)

### Theme 3: Human interaction

The trade-off between the use of digital tools and having human interaction was an important consequence for consideration when using these methods to recruit and retain participants in trials.

‘*Digital recruitment can kind of be seen as a somewhat arm’s length approach, as opposed to a face-to-face discussion. And I’m not saying that you have one without the other, but I have seen some research that suggests people recruited digitally if you like, whilst the recruitment was better the retention was poorer than recruitment via a face-to-face meeting.*’ (ID1 – Trialist)

However, it was felt by the majority of the participants that as long as the appropriate ethical and legal frameworks are adhered to, there is less risk involved and provides reassurance to those taking part in a trial.

‘*I suppose the potential participants might be a bit frightened of the new … once you stop to think about it, they don’t worry, but I think the initial thing is … this bit of a fear of the new … the cyber divide is breaking down and even older people will be able to embrace it.*’ (ID4 – Ethics Committee member)

### Theme 4: Obstacles and risks

For some stakeholder groups, it was felt that a range of approaches and methods should be offered to potential participants, rather than relying on the use of digital tools alone. Therefore, providing flexibility and choice about the use of multi-methods in order to recruit. However, it was also noted how there needs to be some recognition of the speed at which digital technology is evolving and the risks associated to this for both participants and trialists.

‘*I don’t think you could have total digital, and maybe a freedom to say to the person if you need contact … and maybe they just phone up, because that is one to one as well isn’t it? And so having that alongside?*’ (ID01 – Participant representative)

‘ *… The rapidity with which things become obsolete, in terms of digital platforms, is frighteningly rapid. I think that does complicate this space; it makes it more difficult to identify what’s best practice and then replicate it at an industrial scale.*’ (ID11 – Funder)

There was a clear distinction between stakeholders about the value, benefit and influence of existing evidence (or rather lack of this). Charities were more likely to take an evidence-user driven approach, rather than rely on existing academic evidence.

‘ *… We tend to come at it from a “How does the market behave when you ask it to do something?” rather than “We need evidence to do it” because this isn’t trying to introduce a health intervention … recruitment methods for lots of companies like banks and other commercial organisations, there’s a lot to learn from those, so we tend to come at it from that angle … we work out what it is they want to do that doesn’t rely on research evidence … they get back from contributing to and that’s consumer behaviour.*’ (ID12 – Funder)

A number of issues raised by the stakeholder groups focused on staffing issues and the level of expertise required with information technology, the potential cost of developing and setting up of digital tools, the barriers of understanding for funders and ethics committees, the speed at which technologies are advancing and the rapid uptake of usability (e.g. the risk of being obsolete) and how the risk of technical issues can hinder recruitment progression.

‘*We want to wait for the evidence to grow, because it’s quite early days I guess for whether multi-media does enhance information and understanding of potential participants.*’ (ID7 – Ethics member)

‘*Sometimes you drop functionality in your digital tool in order to satisfy everybody … they definitely don’t understand the different levels of security … You’ll get lots of things where they don’t really understand what’s more secure, what’s less secure.*’ (ID2 – Trialist)

### Theme 5: Potential benefits

The overall acceptance of digital technology was clear across all stakeholder groups, despite the lack of or use of evidence to support their use for recruitment and retention of participants. The use of digital tools were seen as having the potential to widen the reach of participant engagement and provide the flexibility for participants to complete data entry.

‘*I can’t see any reason why you wouldn’t want to store the data digitally. The data is going to end up in an electronic format anyway.*’ (ID3 – Participant representative)

‘*People are not bothered about it. I think they’ve got over the botheredliness of it all … it’s accepted that digital tools are extremely useful and, in some instance, the only way you’re going to get your likely participants … I think we’re getting better at it.*’ (ID5 – Ethics Committee member)

‘*You’ve got to demonstrate that you’ve got the expertise to handle the electronic aspect of your research … and have people been offered a choice … the principles of fair consent are the same whether its electronic or face-to-face …*’ (ID4 – Ethics Committee member)

## Discussion

The findings from both CTU survey and qualitative interviews demonstrate the potential role for digital tools in the recruitment and retention of participants in clinical trials. However, the potential benefits from using digital tools are still relatively unknown, despite the frequency of their use. This has important implications not only for research participants and trialists but also for funding organisations and medical research charities. The cost of setting up and maintaining some digital tools is expensive and this can have repercussions for not only trialists but also funders of research. Given the dearth of high-quality evidence to validate or support the use of digital tools, it is imperative that the evidence generated plays particular attention to the nuances particularly identified through the qualitative interviews that ‘not one size fits all’. There is also the need to appreciate that although the Internet can be a potentially valuable tool, there are unchartered ethical considerations for trialists such as the security of personal data, given the new GDPR [21]. Technology is also moving fast, which can make it difficult to identify what is best for a particular group of participants.

As the literature suggests, high-quality evidence is needed to determine the value of digital tools and their potential. Despite this, some trials are already using these technologies to improve trial recruitment and retention with minimal understanding of their effectiveness or appropriateness (lack of evidence to support their use) [6, 9]. By further investigating the use of digital tools for trial recruitment and retention, we will be much better placed to understand their value and benefit for future clinical health research. Only then can we confidently advise on the wider use of digital tools by trialists / researchers and the need for further primary research. This evidence-based approach is critical to counter the ‘apptimism’ (excessive optimism about apps and other digital health tools) that has built up around the use of digital tools in health services research [22].

### Strengths and weaknesses of the study

The main strength of the study was the inclusion of a broad spectrum of stakeholders, using mixed methods to understand their position on the use and experience of using digital tools for the recruitment to and retention in clinical trials [23]. The CTU survey was able to collect views from a range of CTU staff with different job roles. The qualitative interviews comprised five stakeholder groups, including research participant representation, which provided an in-depth understanding from a range of perspectives of how digital tools are viewed. This has important implications for the development of digital tools and their sustainability for use in trials; if participants are unwilling to, or do not see the authenticity / legitimacy of the invitation to participate in a study, problems with recruitment and retention will undoubtedly remain. By using an inductive approach, we were able to gain a valuable insight into what matters to these stakeholders, without influencing their perspective on the use of digital tools.

Although not all CTUs took part in the survey, the responses received provided adequate coverage to begin understanding the types of digital tools used and the variation of their use. Extending the survey duration may have resulted in a higher response rate, although we cannot say with certainty that this would have been the case and the funding was time limited. The qualitative interviews were conducted with a small number of stakeholders (three for each category, with four research participant representatives) and yet the study achieved its recruitment target. By not recruiting to the intended focus group for the participant representative group, it was clear that telephone interviews were the preferred method for these individuals. This has important implications for future research, when considering the use of focus groups with participant representative groups. There is a potential risk of bias due to the sample being purposive and only being limited to randomised trials. However, given the restricted timeframe for completion, the findings from the qualitative interviews provided preliminary data for further consideration.

### Implications

To help fill the gaps in the evidence base we encourage future research to consider conducting more defined and specific systematic reviews on particular digital tools commonly used by CTUs. Future primary research may benefit from greater guidance to improve the evidence base, including liaison with Trial Forge [24, 25], so that assessment of promising digital tools can be evaluated within the NIHR Study within a Trial (SWAT) programme [26]. Possible topics might include: (1) a randomised trial comparing email with social media for recruitment in different age groups, studying the reach across differing socioeconomic groups; and (2) further Delphi research into appropriate tools for people with different disease types and prevention versus treatment. One implication for researchers in this area is that we should study not only the various potential benefits of these tools, but also the potential challenges they raise, as documented in the qualitative study results.

## Conclusion

Our study demonstrates the variety of digital tools being used and how they are seen to be successful by trialists, as well as noting the limited empirical evidence to support their use. Our examples of what constitutes a digital tool (Table 8) will help to inform the NIHR and wider research community about what is currently available and help them identify potential tools to help with recruitment to and retention within their studies.

## Supplementary information

**Additional file 1.** List of questions used in the NETSCC survey.

**Additional file 2.** List of questions used in the UKCRC CTU survey.

**Additional file 3.** Qualitative interview theoretical framework for the interviews.

**Additional file 4.** Preamble for the interview schedules for the five stakeholder groups.

## Data Availability

The data generated or analysed during the current study are included in this published article (and its supplementary information files). Additional information is available from the corresponding author on reasonable request.

## Abbreviations

CTU: Clinical trials unit
CPRD: Clinical Practice Research Datalink
EME: Efficacy and Mechanisms Evaluation programme
EMIS: Formerly known as Egton Medical Information Systems
EPR: Electronic patient records
GDPR: General Data Protection Regulation
GHR: Global Health Research
GRADE: Grading of Recommendations Assessment, Development and Evaluation
HSDR: Health Service and Delivery Research programme
HTA: Health Technology Assessment programme
MRC: Medical Research Council
NETSCC: NIHR Evaluation, Trials and Studies Coordinating Centre
NIHR: National Institute for Health Research
PHR: Public Health Research programme
PPI: Patient and Public Involvement
PICO-M: Population, Intervention, Comparator and Outcomes-Methodology
RCT: Randomised controlled trial
SMS: Short message service
SWAT: Study within a Trial
UKCRC: UK Clinical Research Collaboration

## References

[1] Treweek S, Lockhart P, Pitkethly M (2013). Methods to improve recruitment to randomised controlled trials: Cochrane systematic review and meta-analysis. BMJ Open.

[2] Campbell MK, Snowdon C, Francis D (2007). Recruitment to randomised trials: strategies for trial enrolment and participation study. The STEPS study. Health Technol Assess.

[3] Walters SJ, dos Anjos Henriques-Cadby IB, Bortolami O (2017). Recruitment and retention of participants in randomised controlled trials: a review of trials funded and published by the United Kingdom Health Technology Assessment Programme. BMJ Open.

[4] Raftery J, Young A, Stanton L (2015). Clinical trial metadata: defining and extracting metadata on the design, conduct, results and costs of 125 randomised clinical trials funded by the National Institute for Health Research Health Technology Assessment programme. Health Technol Assess.

[5] Watson JM, Torgerson DJ (2006). Increasing recruitment to randomised trials: a review of randomised controlled trials. BMC Med Res Methodol.

[6] Treweek S, Pitkethly M, Cook J (2018). Strategies to improve recruitment to randomised trials. Cochrane Database Syst Rev.

[7] Gupta A, Calfas KJ, Marshall SJ (2015). Clinical trial management of participant recruitment, enrollment, engagement, and retention in the SMART study using a Marketing and Information Technology (MARKIT) model. Contemp Clin Trials.

[8] Huang GD, Bull J, Johnston McKee K (2018). Clinical trials recruitment planning: A proposed framework from the Clinical Trials Transformation Initiative. Contemp Clin Trials.

[9] Brueton VC, Tierney J, Stenning S (2013). Strategies to improve retention in randomised trials. Cochrane Database Syst Rev.

[10] Kearney A, Daykin A, Shaw ARG (2017). Identifying research priorities for effective retention strategies in clinical trials. Trials.

[11] Köpcke F, Kraus S, Scholler A (2013). Secondary use of routinely collected patient data in a clinical trial: an evaluation of the effects on patient recruitment and data acquisition. Int J Med Inform.

[12] Burrell ER, Pines HA, Robbie E (2012). Use of the Location-Based Social Networking Application GRINDR as a Recruitment Tool in Rectal Microbicide Development Research. AIDS Behav.

[13] Mychasiuk R, Benzies K (2012). Facebook: an effective tool for participant retention in longitudinal research. Child Care Health Dev.

[14] Dizon DS, Sedrak MS, Lewis MA (2018). Incorporating Digital Tools to Improve Clinical Trial Infrastructure: A White Paper From the Digital Engagement Committee of SWOG. JCO Clin Cancer Inform.

[15] Drew CJG, Poile V, Trubey R (2016). Integrating technology into complex intervention trial processes: a case study. Trials.

[16] Bower P, Brueton V, Gamble C (2014). Interventions to improve recruitment and retention in clinical trials: a survey and workshop to assess current practice and future priorities. Trials.

[17] Berger AM, Neumark DE, Chamberlain J (2007). Enhancing recruitment and retention in randomized clinical trials of cancer symptom management. Oncol Nurs Forum.

[18] Crawford S, Hokke S, Nicholson JM (2019). “It’s not black and white”: Public health researchers’ and ethics committees’ perceptions of engaging research participants online. Internet Res.

[19] Köpcke F, Prokosch H-U (2014). Employing computers for the recruitment into clinical trials: a comprehensive systematic review. J Med Internet Res.

[20] Frampton G, Shepherd J, Pickett K, Griffiths G, Wyatt J. OP88 Digital Approaches For Randomized Controlled Trial Recruitment Or Retention: A Systematic Map. Int J Technol Assess Health Care. 2019;35(S1):22–23.

[21] Mulder T (2019). Health apps, their privacy policies and the GDPR. Eur J Law Technol.

[22] Wyatt JC (2015). Fifty million people use computerised self triage. BMJ.

[23] Anguera MT, Blanco-Villaseñor A, Losada JL (2018). Revisiting the difference between mixed methods and multimethods: Is it all in the name?. Qual Quant.

[24] Treweek S (2013). Trial forge: a systematic approach to making trials more efficient. Trials.

[25] Treweek S, Altman DG, Bower P (2015). Making randomised trials more efficient: report of the first meeting to discuss the Trial Forge platform. Trials.

[26] Treweek S, Bevan S, Bower P (2018). Trial forge guidance 1: what is a study within a trial (SWAT)?. Trials.

