# Supplementary material for: Using digital tools in the recruitment and retention in randomised controlled trials: survey of UK Clinical Trial Units and a qualitative study
Source: Trials. 2020 Apr 3;21:304. doi: 10.1186/s13063-020-04234-0 (PMC7118862; doi:10.1186/s13063-020-04234-0)
Supplement: Supplementary file 1 — Additional file 1. List of questions used in the NETSCC survey. [file 13063_2020_4234_MOESM1_ESM.docx]

**Additional File 1.**

NIHR Evaluation Trials and Studies Coordinating Centre survey to research management staff.

Invitation.

Dear Research Manager,

Southampton & Bristol CTUs have been funded by the NIHR Efficient Studies programme to carry out a mapping review and stakeholder survey of the effectiveness of digital tools used to improve the recruitment and / or retention of people in UK RCTs. Such digital tools include
- searches and interactive medical record tools to support clinicians screening participants,
- databases for case finding,
- social media or study websites to publicise a trial,
- email campaigns to engage with the broader public,
- apps or text message programmes to remind trial participants that they are due to complete a study visit or take a study medication etc.,
- interactive websites or apps to retain patients enrolled in trials and help them meet drug or behavioural adherence

As part of this project we would like to gain some understanding about our portfolio to identify commercial, academic or bespoke recruitment or retention tools developed in-house by a project team. So this could be trialists using an already developed tool or testing a tool as part of the management of the trial.

We are therefore requesting your help as a Research Manager to complete a very brief survey (three questions) about such digital tools used in the portfolio that you hold (active project status) or have previously held (the project has now finished). Also, if you are aware of any other projects using digital tools that are not under your responsibility, we would still like to hear about it.

This is a short survey and is designed to be completed in 5-10 minutes.
If you do not have any projects or knowledge about the specified area in question, please still complete the survey and enter NA in the long-answer text boxes. This will ensure we do not contact you and that we have complete coverage of our Research Managers. There are six free text boxes for you to enter the relevant project details (as specified in the survey). If you have more than six examples, please get in touch with me directly ([amanda.blatch-jones@nihr.ac.uk](mailto:amanda.blatch-jones@nihr.ac.uk) or [ajy5@soton.ac.uk](mailto:ajy5@soton.ac.uk) ).

As a way of reassuring you, all the information we collect will be kept confidential and any information shared with the wider project team will be anonymised. We will not be contacting the PI for clarification for those projects you disclose as using digital tools. In addition, the information you provide will not be recorded in any way other than your input to the survey.

Please click on the below link to take you to the online survey.

Can you please complete the survey by 16 February. If this is going to cause an issue for you, please do contact me.

We really appreciate your input into the project, which will help ensure that the effect with digital tools are more widely used. We will be happy to share these findings with you.

On part of the team, many thanks for your help.

| Name: |
| --- |
| Portfolio responsibility for: |
| Please list the reference number of any project that you are aware of which meet the criteria: For each entry provide - 1. Project reference number,  2. What the tool is and,  3. In which document further information can be found.  ***[These questions were repeated for each project entry]*** |
| Date of completion: |
| State whether you would like to receive a summary report of the findings |
