# Supplementary material for: Using digital tools in the recruitment and retention in randomised controlled trials: survey of UK Clinical Trial Units and a qualitative study
Source: Trials. 2020 Apr 3;21:304. doi: 10.1186/s13063-020-04234-0 (PMC7118862; doi:10.1186/s13063-020-04234-0)
Supplement: Supplementary file 2 — Additional file 2. List of questions used in the UKCRC CTU survey. [file 13063_2020_4234_MOESM2_ESM.docx]

**Additional file 2.**

Background for CTU member completing the survey

The Digital Tools study questions are:

1. What digital tools are available that can help identify, recruit or retain people in trials, and what are their associated costs?
2. What characteristics do trialists and CTUs require of these digital tools to be considered useful?
3. What is the evidence about the performance of the most promising tools, and which important evidence gaps need to be filled with primary research to support their greater use?

Digital Tools are defined as computer, phone or tablet-based aids to support recruitment and retention tools by a range of staff involved in trials: e.g. trial managers, people screening patient lists and recruiting or following up participants. We are very keen to identify commercial or academic products or tools developed in-house or bespoke to individual trials, as well as any studies evaluating these (including informal/unpublished studies) or an estimate of their costs. **The survey asks about one or two recruitment tools and one or two retention tools that have impressed you, and about one of either kind that has problems**.

Examples of Digital Tools include:

- Database searches for offline case finding, eg. via CPRD
- Tools that flag up a patient as eligible for a study during a consultation, using the electronic patient record, e.g. point of care trials
- Study websites, social media, email or text message campaigns to publicise a trial to potential participants or trial directories e.g. ClinicalTrials.gov
- Apps or text messages for participants as reminders for making a trial observation, visit or medication

We expect that there are other Digital Tools and we would like to hear about them.

Please don’t focus on the following:

- A word processor to format a poster advertising the trial
- A spreadsheet charting trial recruitment over time
- A sample size calculator for developing the protocol or grant application
- A trial simulator of trial selection criteria on recruitment rates before the trial starts
- Databases, spreadsheets etc. for capturing data from those enrolled in a trial, e.g. MACRO
- External recruitment agencies who may use any of the above to improve recruitment rates

The outline of the overall project is shown below. Phase 1 has been completed and your answers to the survey are key to Phase 2.


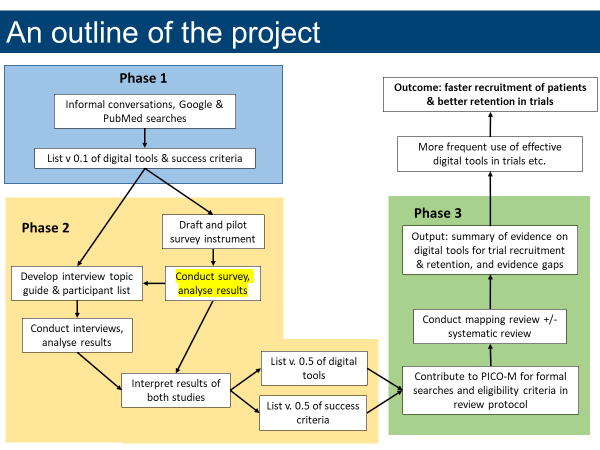


**Survey Questions**

| **About you** | |
| --- | --- |
| Name of your CTU |  |
| Name of the person completing this form |  |
| Job title |  |
| Date you completed form |  |
| Email (for queries) |  |
| **Recruitment and retention tools your CTU has experience of** | |
| Please list all digital tools your CTU has had experience of in relation to recruitment |  |
| Please list all digital tools your CTU has had experience of in relation to retention |  |
| **About the first digital recruitment tool, and how it is used in your CTU** | |
| What is the name of the tool used ? |  |
| What is the main recruitment barrier for which this tool is a solution ? |  |
| Source of the tool *(please select)* | Commercial product / Academic (eg. student project) / in house bespoke CTU software / Other (*please specify*) |
| Main disease area tool is used with (eg. cancer, asthma, surgical trials) |  |
| Is the tool specific to the needs of a care group (children, frail elderly, adults with learning difficulties etc.) | If yes, which group: |
| Is the tool currently in use in your CTU? | Yes/ No |
| If Yes, for how long has the tool been used, and what is the main reason behind its continued use? | _______ months / years  Main reason: |
| If no, for how long was the tool used and what was the reason behind stopping using it? | _______ months / years  Main reason: |
| Is the tool just used for one study or has it be used / configured for more than one study? | One study / >1 study |
| If used for more than one study, for how many has it been used ? | ____ studies |
| If known, approximately how many hours does it take configure the tool for a new study? | unknown  ____ hours |
| Subjectively, how easy does it feel to configure this tool ? | Need to get an expert to do it for us / we need to go on a course / need to study online material eg. videos as well as manual / just need to read manual / just refer to manual if problems / no need to refer to any materials |
| **About the cost, benefits and side effects of this first digital recruitment tool** | |
| Is / was this tool considered to be effective | Yes, very / yes, partly / possibly, to some extent / not really / not at all |
| If Yes very or Yes partly: in what way is / was it effective, and for whom ? | In what way ________  To who ________ |
| Do you have any data to back up this view? | Yes/ No/ Unsure |
| If yes, please let us know how to access this data (web link, contact person etc.) |  |
| Have you noticed or measured any side effects of using this tool (eg. more patients recruited but more drop outs; inappropriate patients recruited; recruitment staff find it burdensome): | Yes/ No |
| If Yes, please describe these side effects | ________ |
| What is your evidence for this? *(please select)* | informal staff views / formal staff survey / lab test of tool accuracy / field test of impact on recruitment or retention rates / Other *(please specify)* |
| If possible, please provide an estimate of the increase in efficiency when using the tool (as a %) in a part of the trial process. What part of the overall trial process does this form (as a %) ? | ___ % of the process  This process is ___ % of the whole trial process |
| Would you be willing for one of our researchers to interview you by phone or Skype to discuss this tool and its usage / impact / side effects? | Yes/ No |
| If Yes please specify preference for phone or email and provide details as appropriate. | Email or phone details |
| *Please attach a copy of any relevant internal reports or any external publication evaluating the tool* | |
| **About the second digital recruitment tool (optional) – as above** | |
| **About the first digital retention tool – as above** | |
| **About the first digital retention tool (optional) – as above** | |
| **About either kind of tool that has caused problems (optional)** | |
|  | |
