# Supplementary material for: Using digital tools in the recruitment and retention in randomised controlled trials: survey of UK Clinical Trial Units and a qualitative study
Source: Trials. 2020 Apr 3;21:304. doi: 10.1186/s13063-020-04234-0 (PMC7118862; doi:10.1186/s13063-020-04234-0)
Supplement: Supplementary file 3 — Additional file 3. Qualitative interview theoretical framework for the interviews. [file 13063_2020_4234_MOESM3_ESM.docx]

**Additional file 3.**

**Using digital tools for recruitment and retention in trials - stakeholder perceptions from semi-structured interviews**

**Theoretical framework for data collection and analysis**

**Stakeholders**  **Concepts Topics**

Benefits

To the study

- Accuracy
- Easy to use
- Effective
  - Appropriate patients
  - Wider range of patients
  - More patients
  - Patients who stay in

To the patient

- No need to travel to hospital
- Less time commitment
- Easy to use

Risk or harm

To the study

- More drop out
- Privacy issues
- Costs
- False positives

To the patient

- Lack of technical skill
- Unable/unwilling to engage

Systems and processes

- Seeking research funding
- Appropriate ethical principles
- Involvement of patients and the public
- Privacy
- Confidentiality
- Data protection / GDPR

About the digital tool

- Evidence of effectiveness
- Quality - what makes it good or bad
- Type of digital tool

Concerns about digital tools

- Generic
- Specific (to a patient or professional group)

No concerns about digital tools

- An integral part of our world
- Any risks are no different from non-digital methods

Research funding bodies

*

Ethics Committees and Health Research Authority

*

Trialists in Clinical Trials Units (secondary care)

*

Research practitioners in primary care

*

Research participant/patient representative
