# Supplementary material for: Using digital tools in the recruitment and retention in randomised controlled trials: survey of UK Clinical Trial Units and a qualitative study
Source: Trials. 2020 Apr 3;21:304. doi: 10.1186/s13063-020-04234-0 (PMC7118862; doi:10.1186/s13063-020-04234-0)
Supplement: Supplementary file 4 — Additional file 4. Preamble for the interview schedules for the five stakeholder groups. [file 13063_2020_4234_MOESM4_ESM.docx]

**Additional file 4.**

**Digital tools study – Interview framework for all interviewees**

Thank you for agreeing to participate in this research interview

Regarding the Participant Information Sheet we sent to you, did you have any questions about what we said about the study?

So just to clarify before we start:

**Purpose of the study:**

To explore the use of digital tools to support the recruitment and subsequent retention of patients in clinical trials.

**Definition**:

By ‘digital tools’ we mean things like searches and interactive medical record tools to support clinicians in screening participants for eligibility for studies or for recruitment of patients.

For eligibility, examples include trial websites, social media and email campaigns to engage with the broader public.

For retention of study participants examples include emails, interactive websites, text messages or apps.

**Why are we doing this study?**

A systematic review is needed but at the moment we don’t really know what criteria stakeholders would use to judge the quality of digital tools, nor do we know how rigorous the studies of digital tools need to be to provide this evidence. So, we need to carry out this qualitative study first to provide information about the outcomes and methods that are relevant for the systematic review. The qualitative study includes interviews with stakeholders like yourself.

**Who are the stakeholders we are interviewing?**

Trialists, primary care researchers, research funders, ethics committee/HRA representatives and study participants (through focus groups).

**Why you?**

We are asking you in your capacity as [INSERT ROLE trialists, primary care researcher, research funders, ethics committee/HRA representatives] to give us your opinions about using digital tools.

**Consent and confidentiality**

Before we start the recording I will ask you to confirm that you agree with the following statements:

- Your participation is voluntary and you may withdraw at any time before the interview
- You give your consent to participate and for the audio-recording of the interview
- You have been offered the opportunity to ask any questions about the study
- Your role in this research will remain confidential. The audio file from the recording and the interview transcript will be stored securely in a password protected folder and accessed only by the researcher/s undertaking the interviews
- The final report and any subsequent publication will not contain any identifiable material
- You give your consent for anonymised quotes to be used in these reports.

**Digital tools in trials recruitment and retention**

**Interview framework – version 3 1/6/18**

**Interview prompts for Research Funders**

Your role

General

- Ethics
- Funding
- PPI
- Data protection, GDPR regulations
- Confidentiality
- Privacy

**Interview prompts**

What digital tools have you heard of?

What features would/do you look for?

What criteria would you use to decide whether it was a good tool or not?

What do board or panel members feel if they come across digital tools they’ve never heard of?

Do you think about what evidence is needed for digital tools?

What would put off the board about funding a proposal using digital tools?

Has the board funded or would it fund a Study within a Trial (SWAT) if it used digital tools? Which one? What would put the board off?

Would your view as a funder change if a proposal included the use of digital tools?

**Digital tools in trials recruitment and retention**

**Interview framework – version 2 29/3/18**

**Interview prompts for Ethics committee / HRA**

Your role

General

- Ethics
- Funding
- PPI
- Data protection. Did the new GDPR regulations have any influence?
- Did you have to think about any confidentiality issues?
- Privacy
- Will you use this for all your trials?

**Interview prompts**

Does the use of digital tools in research proposals pose any special issues for the committee?

Do you think about what evidence is needed for digital tools?

For example, is the use of digital tools seen to:

- put participants under stress?
- create privacy issues?

What digital tools have you heard of?

Are some types of digital tool better than others?

What features would/do you look for?

What criteria would you use to decide whether it was a good tool or not?

*[Once we have an understanding of the criteria that are used by CTUs for determining the usefulness of a digital tools, we will ask an Ethics Committee representative their thoughts about these]*

**Digital tools in trials recruitment and retention**

**Interview framework – version 2 29/3/18**

**Interview prompts for trialists (e.g. Principal Investigators) and Research Nurses/Practitioners**

Your role

General

- Ethics
- Funding
- PPI
- Data protection. Did the new GDPR regulations have any influence?
- Did you have to think about any confidentiality issues?
- Privacy
- Will you use this for all your trials?

**Interview prompts**

What do you think about using digital tools:

- When designing a study?
- When applying for funding?

What do you want to gain from using digital tools?

What digital tools do you use or have you heard of?

Are some types of digital tool better than others?

What features would/do you look for?

What criteria would you use to decide whether it was a good tool or not?

What outcomes are you looking for in using digital tools? Increase in number of enquiries?

What specific features are necessary for retention tools? Recruitment tools?

What evidence of success would you look for when deciding whether to use a particular tool?

What kind of evidence/level of evidence would convince you that the tool would work?

What tools have you tried using which didn’t work? What was it that didn’t work? What would have made it more effective for you? Why didn’t you like it?

Do you know about any of the following for help with recruitment and retention in trials:

- Database tools off-line?
- Websites?
- Short message service (SMS or text)/email?
- Social media?
- Pop-up on the electronic patient record (EPR)?

*Further prompts about specific tools from the list immediately above*

- Have you ever used one of these tools?
- If you think this would be a useful tool what would be the useful features of this tool?
- What do you think would be the difficulties?
- What would need to happen to make this tool more useful for you?

**Digital tools in trials recruitment and retention**

**Interview framework**

**Primary care staff**

Your role

General

- Ethics
- Funding
- PPI
- Data protection. Did the new GDPR regulations have any influence?
- Did you have to think about any confidentiality issues?
- Privacy

**Interview prompts**

What do you think about using digital tools:

- When designing a study?
- When applying for funding?

What do you want to gain from using digital tools?

What digital tools do you use or have you heard of?

Are some types of digital tool better than others?

What features would/do you look for?

What criteria would you use to decide whether it was a good tool or not?

What outcomes are you looking for in using digital tools? Increase in number of enquiries?

What specific features are necessary for retention tools? Recruitment tools?

What evidence of success would you look for when deciding whether to use a particular tool?

What kind of evidence/level of evidence would convince you that the tool would work?

What tools have you tried using which didn’t work? What was it that didn’t work? What would have made it more effective for you? Why didn’t you like it?

Do you know about any of the following for help with recruitment and retention in trials:

- Database tools off-line?
- Websites?
- Short message service (SMS or text)/email?
- Social media?
- Pop-up on the electronic patient record (EPR)?

*Further prompts about specific tools from the list immediately above*

- Have you ever used one of these tools?
- If you think this would be a useful tool what would be the useful features of this tool?
- What do you think would be the difficulties?
- What would need to happen to make this tool more useful for you?

**Digital tools in trials recruitment and retention**

**Patient representative interview framework**

**Interview prompts**

Do you have experience of being a study participant when digital tools were used for recruitment or retention of participants?

If you haven’t had experience of using a digital tool as a trial participant we’d like you to try to imagine what it would be like to be a participant using digital tools which have been designed to help researchers with recruitment and retention of study participants.

What features of a digital tool would you want to have access to?

What criteria would make it a good tool or not from your perspective?

For recruitment and retention how would you feel about the use of:

- Text messaging
- Emails
- Interactive websites
- Social media
- Apps

How would these be helpful to you?

What would make them unsuitable for you to use?

What would make you want to use them?

What would put you off using them?

For those who have experience of using digital tools in trials

- What digital tools did you use?

Do you know about any of the following for help with recruitment and retention in trials:

- Websites?
- Short message service (SMS or text)/email?
- Social media?
- Pop-up on your patient record in the GP practice (Pop-up on electronic patient record (EPR)?
